# Ultrastructure of the antennal sensillae of male and female peach fruit fly, *Bactrocera zonata*

**DOI:** 10.1093/jis/14.1.45

**Published:** 2014-01-01

**Authors:** Azza A. Awad, Nashat A. Ali, Hend O. Mohamed

**Affiliations:** 1 Zoology Department, Faculty of Science, Assiut University, Egypt; 2 Plant Protection Res. Institute, Agricultural Research Center, Giza, Egypt

**Keywords:** antennae, funicular sensilla, scanning electron microscopy

## Abstract

Antennal morphology and funicular sensillae of male and female peach fruit flies, *Bactrocera zonata* (Saunders) (Diptera: Tephritidae), were studied with scanning electron microscopy (SEM). This study focused on the sensillae found on the antennal segments (scape, pedicel, and flagellum or funiculus that bears the arista) of *B. zonata* . Antennal segments of females tended to be larger than those of the males. The first two antennal segments, scape and pedicel, were heavily covered with microtrichia and bear bristles. Numerous microtrichia as well as trichoid (I, II), basiconic, clavate, and coeloconic sensillae were observed on the funiculus. SEM studies showed some differences in size and also in position of some sensillae on the antennae of the females of *B. zonata* . The sensillae found on the funiculus, such as trichoid and basiconic sensillae, were significantly larger in females.

## Introduction

The peach fruit fly, *Bactrocera zonata* (Saunders) (Diptera: Tephritidae), is considered one of the most destructive fruit pests in several regions of the world (Drew 1989). It has been recorded on more than 50 cultivated and wild plant species (Kapoor and Agarwal 1982; White and Elson-Harris 1992; EPPO 2003). It attacks ripe fruits and inflicts damage to the fruits directly (through oviposition punctures and subsequent larval feeding on pulp) or by creating blemishes on fruits, which limit marketing possibilities (especially export of fruits) (Aluja et al. 1996; EPPO 2003). The infestation may reach up to 50% in summer crop of guava in Pakistan. The fruit attacked by this pest become malformed, misshaped, under-sized, and rotted inside (Atwal 1976).

Antennae of insects play great roles in their chemical communication, host preference, and chemoreception of sex pheromones, which are of primary importance in pest management strategy. Insect’s antennae have various types of sensilla with different functions that play important roles in various behaviors during adult life. Antennal sensillae are important sensory receptors used in host location and discrimination behaviors (Schneider1964; Ochieng et al. 2000). The antenna is the major channel of sensory input, including receptors for volatile odors and pheromones, contact chemoreception, water vapor, carbon dioxide, sound perception, and touch (Ehmer and Gronenberg 1997; Renthal et. al. 2003). The feeding and reproductive behavior in tephritid fruit flies depends greatly on the chemical stimuli they receive and process (e.g., pheromones and plant volatiles, such as methyl eugenol). The primary structures involved in the initial reception of semiochemical information are the antennal chemoreceptors (Rice 1989; Keil 1999). Therefore, in order to achieve successful control of agricultural pests using synthetic sex pheromones, it is essential to have a better understanding of the peripheral sensory structure involved in the perception of pheromones, the antenna being the primary sensory structure.

The antennal morphology and types of sensilla have been described for several tephritid species of economic importance. Among these are *Bactrocera* ( *Dacus* ) *oleae* Gmelin (Hallberg et al. 1984); *Bactrocera* ( *Dacus* ) *tryoni* Froggatt (Gianakakis and Fletcher 1985; Hull and Cribb 1997); *Ceratitis capitata* Wiedemann (Levinson et al. 1987; Mayo et al. 1987; Dickens et al. 1988; Bigiani et al. 1989); *Anastrepha ludens* Loew, *Bactrocera* ( *Dacus* ) *cucurbitae* Coquillet, *Bactrocera* ( *Dacus* ) *dorsalis* Hendel (Dickens et al. 1988), *Eurosta solidaginis* Fitch (Vasey and Ritter 1987), *Anastrepha serpentine* Wiedemann (Castrejón-Gomé 2006), and *Bactrocera tau* Walker *, Bactrocera cucurbitae* Coquillett *, Bactrocera minax* Enderlein, *Bactrocera diaphora* Hendel, *and Bactrocera scutellata* Hendel (Hu et al. 2010). However, very little is known about *B. zonata* .

According to the various types of insect’s sensilla, their positions, and functions, one can differentiate between the different species or even between morphs of the same species (dimorphism). Generally, sensillae are considered as the main communication system in insects, and with them individuals locate their partners and host plants. According to their functions, the antennal sensillae may be divided into chemoreceptors, mechanoreceptors, and thermo-/hygroreceptors (Zacharuk 1985; Clements 1999). Moreover, sensilla can play an important role in insect control (biological or chemical control). Therefore, the studying of chemoreceptor sensilla can be used in control by using insecticides that block the function of these sensilla. The aim of this paper is to investigate the antennal sensory structures of peach fruit fly ( *B. zonat* a), with the goal of identifying and characterizing sensillae types involved in chemoreception. We present here the first examination of the morphology, abundance, and distribution of antennal sensillae in both male and female *B. zonata* . It is anticipated that this study will facilitate future research on the electrophysi-ology and neurobiology of olfaction in peach fruit flies.

## Materials and Methods

### Collection and rearing the flies

Larvae of *B. zonata* were obtained from infested mango fruit ( *Mangifera indica* L. (Sapindales: Anacardiaceae)), which were collected from the field and placed in plastic plates containing sand at the bottom. The jumping larvae, which pupated in the sand, were collected and transferred to adult rearing cages until adult emergence. The newly emerged flies were separated by sex, provided with adult food consisting of sugar mixed with hydrolyzate protein (yeast) at a ratio of 3:1 by weight, and then introduced to the microscopy studies.

### Scanning electron microscopy

Both male and female adult flies (seven days old) were picked to be examined for ultrastructure and morphological characters by scanning electron microscopy (SEM). Scanning electron microscopy was performed as described by Azza (1999). The four following steps were performed:

1) **Fixation.** Two fixation techniques were used: (1) glutraledehyde + osmium tetroxide fixation (using critical point drying). This first fixation was by using GTA technique, but it was not the proper fixative for that specimen.

(2) Khal’s solution fixation (using freeze-dry-ing). In this study Khal’s solution was used as a fixative and it was prepared as follows: 30 mL 95% EtOH + 12 mL formaldehyde + 4 mL glacial acetic acid + 60 mL distilled water. Specimens were ready for fixation directly after mixing these components. Fixation by Khal’s solution lasted 7–8 days.

**2) Dehydration** . After fixation, the fixative was washed by three washes in the same buffer vehicle as used for the fixative. Ethanol is the most widely used dehydration agent. After secondary fixation, specimens were left in a series of ascending alcohols (30% for 2 hr, 50% for 2 hr, 70% for 2 hr, 90% with two changes for 2 hr, and 100% with three or four changes for 2 hr) in order to eliminate the small amount of water remaining in the tissue. Then excess alcohol was drained off, and specimens were put in amylacetat for 1–2 days.

**3) Drying.** Because of their hard outer shell, the specimens were air-dried.

**4) Final mounting.** After a specimen was dried, it was mounted on an SEM holder called a specimen stub. The surface of the stub was as smooth and free of structure as possible in order to prevent confusing backgrounds with sticky-taps (adhesive). The specimen was then carefully placed onto the adhesive, and clean air was used to press it into the adhesive. The specimen holder was labeled from the underside with a permanent marker pen and stored until was needed for sputter coat-ing. The specimen was then coated with gold film with 150 Aº thickness using an JFC-1100E sputtering device (JEOL, www.jeol.com ) for 2–3 minutes. The specimen was examined using a JEOL 5400LV scanning electron microscope. Identification of sensilla was carried out according to Snodgrass (1944) and Zacharuk (1985).

### Statistical Analysis

Sensilla on the dorsal surfaces of the antennae of *B. zonata* were identified and measured. Measurements (µm) obtained from photomi-crographs of at least 10 individual sensilla of the same type were used to calculate the means. Data obtained on the different segments of the antenna on male and female *B. zonata* were analyzed using a *t* -test with SPSS 12.0 for Windows (IBM, www.ibm.com ) to determine any significant differences ( *P* < 0.05).

## Results

### Morphology of the fly antennae

**Figure 1.**
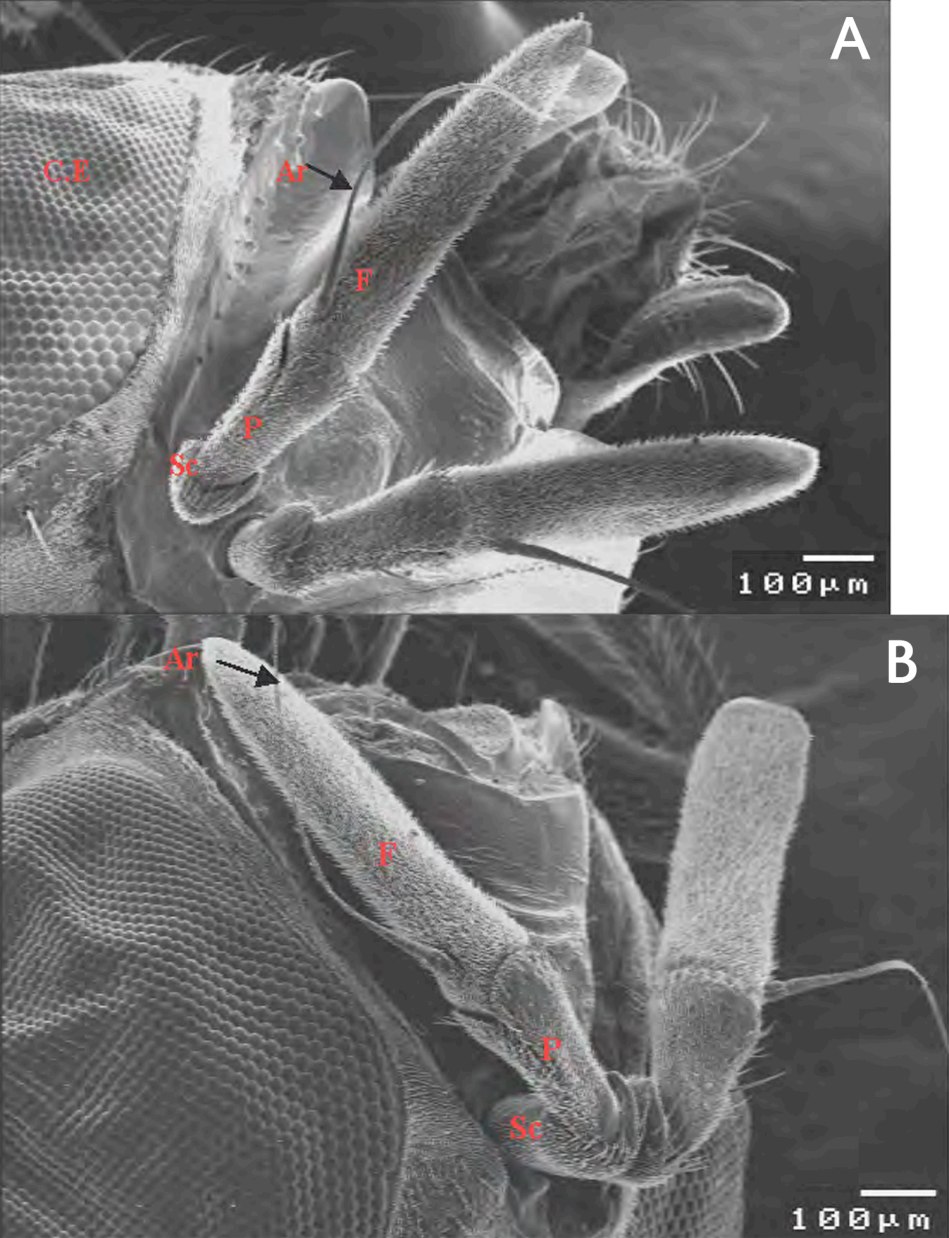
Scanning electron micrograph of the antennal segment of male (A) and female (B) *Bactrocera zonata.* Sc: scape; P: pedicel; F: funiculus; Ar: aristae. High quality figures are available online.

The antennae of peach fruit fly, *B. zonata* , are situated in a frontal depression between compound eyes (the antennal fossa). The antenna is composed of three segments, the scape, pedicel, and funiculus. The scape (short basal segment, semicircle shape) attaches to the pedicel and is movable with it to allow the movement of antenna. Both the scape and the pedicel are heavily covered with microtrichia and bear bristles. The funiculus (third antennal segment) is unsegmented flagellum, while the arista was found on the dorso-proximal end of the funiculus ( Figure 1A , B ). The total length of the antenna and the length of each segment are shown in Table 1 . The antennal segments of females (~1000 µm) tended to be larger than those of males (~846.15 µm)

**Table 1.**
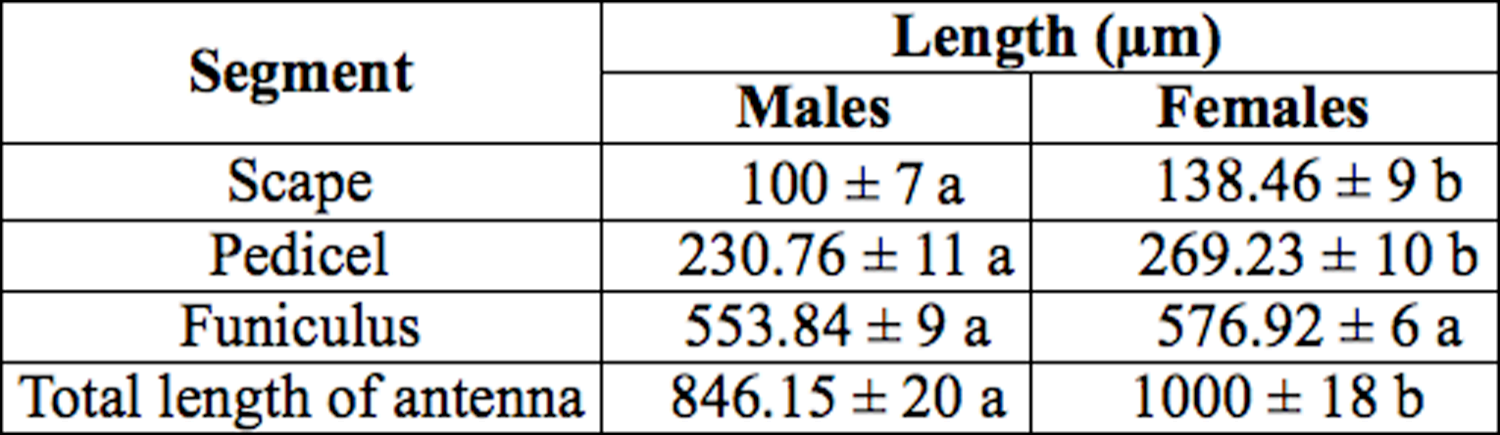
Measurements (μm) of antennae of male and female peach fruit fly, *Bactrocera zonata.*

Values are mean ± standard error; n = 5. The values for each segment within a parameter by sex followed by the same letter are not significantly different (Student’s *t* -test, *P* > 0.05).

**Figure 2.**
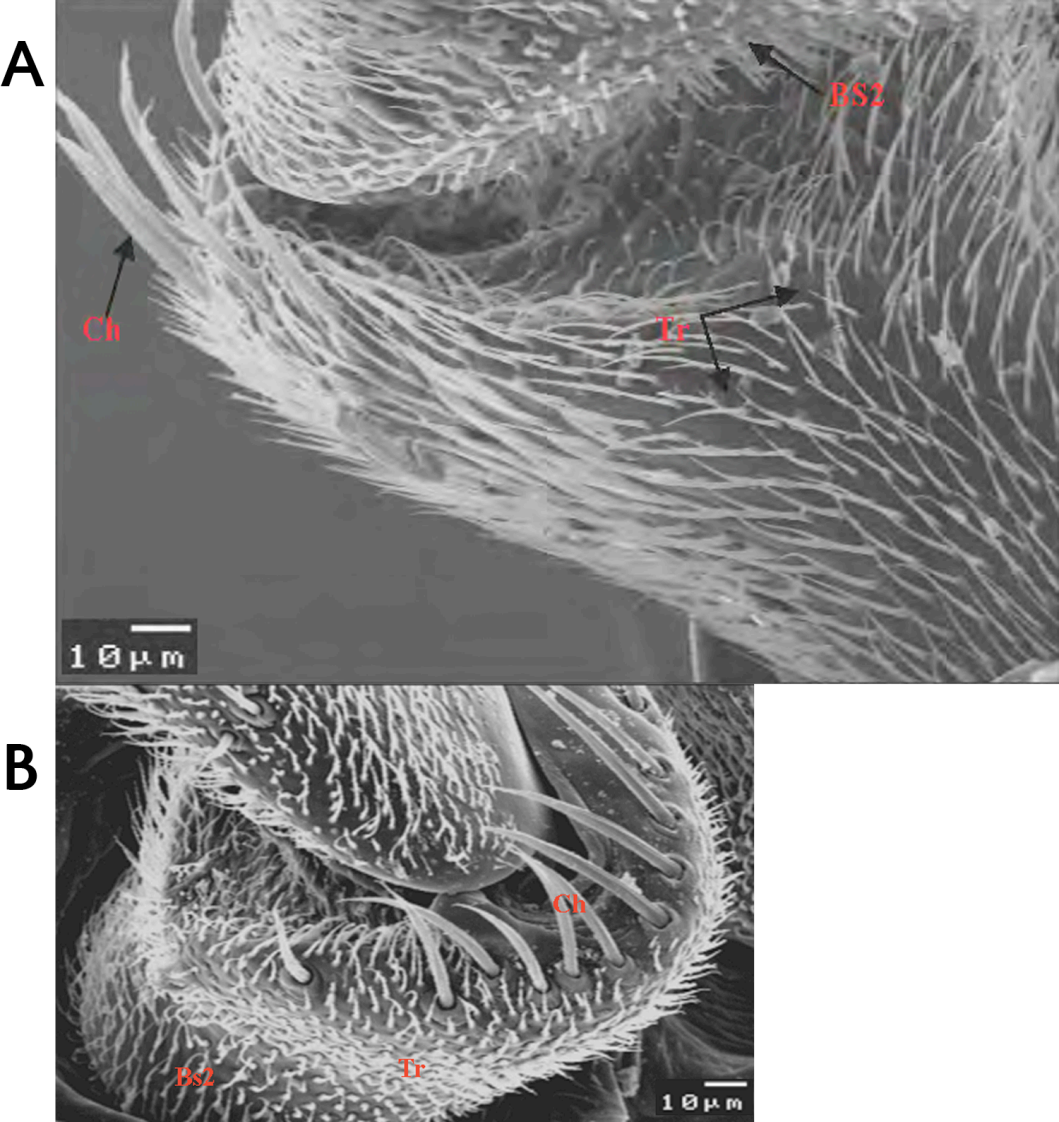
Scanning electron micrograph of the scape of male (A) and female (B) *Bactrocera zonata* showing different types of sensilla. Tr: trichoid sensilla; BsII: basiconica sensilla type 2; Ch: cheatica sensilla. High quality figures are available online.

**A) The scape** (Sc; basal antennal segment). The scape is a very narrow area that attaches the antennae to the head capsule. It is reinforced by some bristles and carries the following sensilla ( Table 2 , Figure 2A , B ):

**Table 2.**
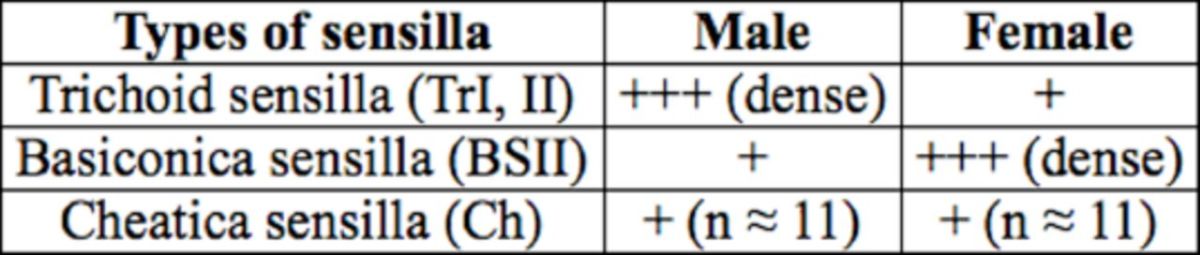
Different types of sensilla observed on the scape of male and female peach fruit fly, *Bactrocera zonata.* + to +++ indicate relative numbers of sensilla.

1)Trichoid sensilla (TrI, II): Scattered over the surface area of the scape in both sexes, but in males more densely distributed than in of females.
2)Basiconica sensilla (BSII): Scattered over the surface area in both sexes, with a swollen base and short neck shaft. More densely distributed in females than in males.
3)Cheatica sensilla (Ch): One row of bristle-like structure, the cheatica sensilla run in the middle area of the scape. It has a stout and very long shaft, which arises from a rounded cavity in the surface of the cuticle.

**Figure 3.**
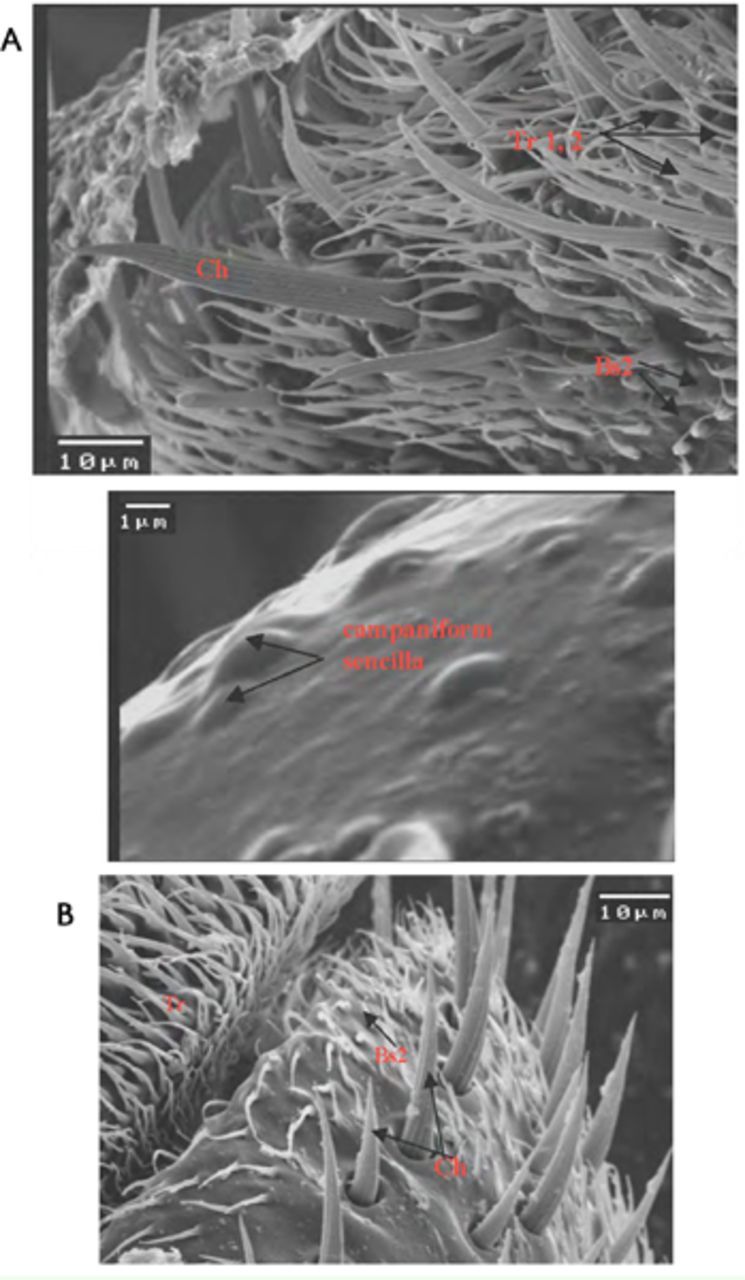
Scanning electron micrograph of the pedicel of male (A) and female (B) *Bactrocera zonata* showing different types of sensilla. TrI, II: trichoid sensilla type 1, 2; BsII: basiconica sensilla type 2; Ch: cheatica sensilla. Campaniform sensilla only on the pedicel of male. High quality figures are available online.

**B) The pedicel** (P; second antennal segment). The pedicel is a cone-like structure that measures ~284.5 µm in its maximum length, slightly longer than the scape, movable with scape to allow the movement of antenna. It isreinforced and fringed with many types ofsensilla ( Table 3 , Figure 3A , B ). The totalnumber of sensilla observed in males wasgreater than in females. Numerous microtrichia as well as trichoid (TrI, II), cheatica, and basiconica (II) sensilla were observed on the pedicel.

**Table 3.**
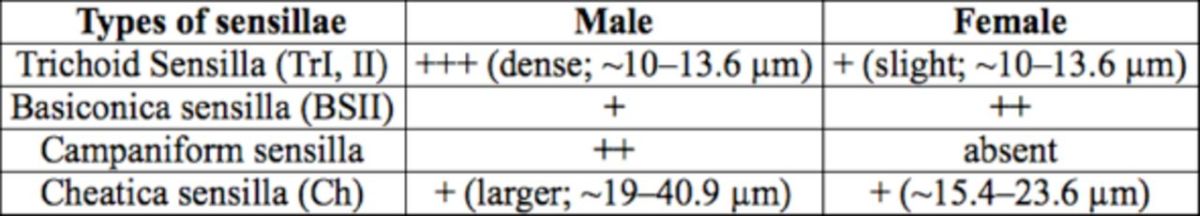
Different types and measurements (μm) of sensilla observed on the pedicel of male and female peach fruit fly, *Bactrocera zonata* , derived from at least ten measurements of each sensillar type for each sex. + to +++ indicate relative numbers of sensilla.

1) Trichoid sensilla (Tr I, II): The most conspicuous sensilla observed in both sexes, but thicker in males than in females.
2) Basiconica sensilla (BSII): Basically trichoid hairs that are much reduced in length and changed in form to be swollen at the base with short neck. Found in both sexes.
3) Campaniform sensilla (dome-like structure): Found in males but absent in females.
4) Cheatica sensilla (Ch): Long, fluted spines or bristles that arise from a depression of the surface of the cuticle. Found at the periphery of the pedicel near the base of the funicule. Found in both sexes, but larger and thicker in males than in females.

**Figure 4.**
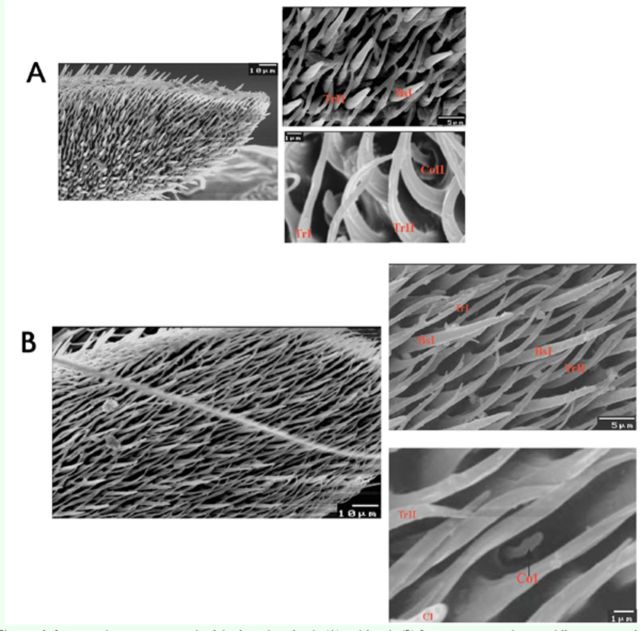
Scanning electron micrograph of the funiculus of male (A) and female (B) *Bactrocera zonata* showing different types of sensilla. TrI, II: trichoid sensilla type 1, 2; BsI: basiconica sensilla type 1; Cl: clavate sensilla; CoI, II: coeloconica sensilla type 1, 2. High quality figures are available online.

**C) The funiculus** (third antennal segment): The funiculus is the most important antennal segment. It is an elongate and unsegmented flagellum. A large protruding arista extends from the superior edge of the outer surface of the funiculus. The third antennal segment of females (~576.92 µm) tended to be larger than that of males (~553.84 µm). Numerous sensilla were found. It is densely covered with microtrichia, which gradually diminished in density from the base to the tip. Four major types of sensilla (trichoid, basiconica, clavate and coeloconica sensillae) were observed on the flagellum (funiculus) of male and female *B. zonata* . All sensillae were oriented in a direction to the tip of antenna, giving the flagellum a velvety appearance. The funicular sensilla observed in males was greater than in females ( Table 4 , Figure 4A , B ).

**Table 4.**
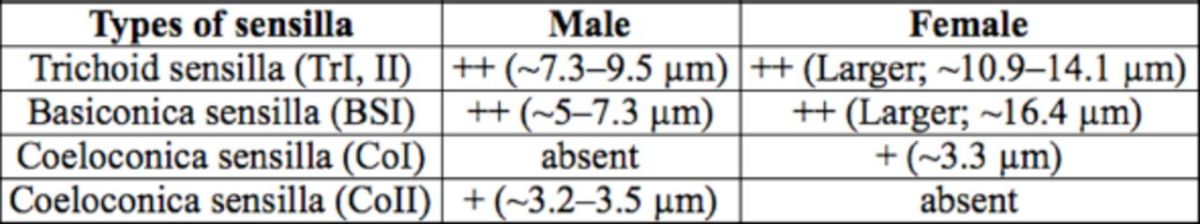
Different types of sensilla observed on the funiculus of male and female peach fruit fly, *Bactrocera zonata* , derived from at least ten measurements of each sensillar type for each sex. + to +++ indicate relative numbers of sensilla.

1)Trichoid sensilla (Tr I, II): The first type of trichoid sensilla (TrI) is the most numerous type found on the flagellum. Densely distributed over the dorsal surface, but rarely found on the proximal part of the ventral surface. The second type (TrII) is usually slightly curved, thin walled sensilla. Less numerous than the first type (I). Trichoid sensillae longer in females than in males, but thicker in males.
2)Basiconica sensilla (BS I): Basiconica sensillae were well distributed in the floor of the funicular surface. Characterized as digitiform (finger-like) with a rounded point and a smooth surface. Basiconic sensilla showed great variation in length, larger in females than in males.
3)Clavate sensilla: This type of sensilla is not a very common receptor type. Localized on the proximal end of the funiculus, close to the pedicel. Clavate sensilla similar to the basiconic sensilla but shorter and club-like. Found in both sexes.
4)Coeloconica sensilla: Coeloconic sensillae are the shortest and fewest sensillar types found on the flagellum of males and females. Scattered irregularly on the whole surface and arise from a depression of the integument or cavity called “sacculus.” Sacculus has a single opening with an irregular rounded margin at the cuticle surface. Males contain coeloconica sensilla type (II) (CoII), which are curved, while females have coeloconica sensilla type (I) (CoI).

## Discussion

The antennae of *B. zonata* were very similar in terms of their general structure to those of other fruit flies studied, such as *A. ludens* , *C. capitata* , *D. cucurbitae* , *D. dorsalis* , and *A. serpentine* (Levinson et al. 1987; Dickens et al. 1988; Castrejón-Gómez 2006), which are composed of three segments, although the size of the various sensillar types varies from species to species. The significant conspecific morphometric difference was in the total length of the antennae of male and female *Dacus* species. However, the greater length of the female antenna might suggest females have more sensillae than males, as is true of the Queensland fruit fly, *D. tryoni* (Giannakakis and Fletcher 1985).

This study showed that in *B. zonata* , both sexes had three distinct types of the sensilla on the scape: trichoid, basiconic II, and cheatica. These sensillae were similar to *L. babiyari* (Ehab 2008). Sukontason et al. (2004) recorded only one type of trichoid sensillae on the scape of *P. dux* , while the basiconic type was absent. Also, Arzuffi et al. (2008) reported that both sexes of *T. curvicauda* had only trichoid sensilla. The results indicated that the pedicel had two types of trichoid (trichoid sensillae are mechano-sensitive, chemo-sensitive, and olfactory receptors and could be daully mechano-/chemoreceptors), basiconic II, and cheatica sensilla (which function as mechanoreceptors) on both sexes. Also, the male only had campaniform sensilla (dome-like structure), which are mechanoreceptors; these sensilla may be because males are strongly attracted to and compulsively feed on methyl eugenol, while no females are ever attracted to or captured in methyl-eugenol -baited clear-traps (Tan and Nishida 2012). However, three types of trichoid sensillae (Tr I, II and IV) were found on the pedicel of *L. babiyari* (Ehab 2008). Sukontason et al. (2004) reported the presence of only one type of trichoid sensillae on *P. dux* . However, Manoj and Sofian-Azirun (2002) found only sensilla chaetica (ch) on the scape and pedicel of *B. caraznbolae.* Trichoid sensillae function as a mechanoreceptors in numerous insects, as stated by Fernandes et al. (2002) for *Dermatobia hominis* , by Merivee et al. (2002) for *Bembidion properans* , by Renthal et al. (2003) for *Solenpsis invicta* , and by Ochieng et al. (2000) for *Microplitis croceipes* .

Four morphologically distinct types of the sensillae were found on the funiculus: trichoid (I, II), basiconic (I), clavate, and coeloloconic. These sensilla are similar to the ones reported in other species of tephritids (Hallberg et al. 1984; Gianakakis and Fletcher 1985; Levinson et al. 1987; Mayo et al. 1987; Vasey and Ritter 1987; Dickens et al. 1988; Bigiani et al. 1989; Hull and Cribb 1997; Castrejón-Goméz 2006, Chen and Fadamiro 2008). Similar findings of the two types of trichoid sensillae present on the antenna were reported in *Calliphora erythrocephala* (Berned 1985; Kuhbandner 1984); in *Chrysomya penguins* , *C. megacephala* , *C. rufifacies* , and *Lucilia cuprina* by Sukontason et al. (2004); and in *Cochliomyia hominivorax* by Fernandes et al. (2004). However, Giangiuliani et al. (1993) mentioned that *Trichopoda pennipes* completely lacked trichoid sensillae.

The results of our study showed that trichoid and basiconic sensilla of the females were significantly longer than those of the males. This difference may be related to the function of the female’s chemoreceptors. On the other hand, this phenomenon could be related to the sex pheromones received by the antennae of males. Also, similar findings were reported on the basiconic (Bs I) on the funicle of *P. nigrolineata* by Rahal et al. (1996); in *T. pennipes* by Giangiuliani et al. (1993); in *Calliphora erythrocephala* by Kuhbander (1984); in *Chrysomya penguins* , *C. megacephala, C. rufifacies* , and *Lucilia cuprina* by Sukontason et al. (2004); and in *Cochliomyia hominivorax* by Fernandes et al. (2004). In contrast, Sukontason et al. (2008) noticed the presence of two types of large aporous and small porous basiconical sensillae on the funicle of *C. penguins* .

Sensilla are considered the main communication system in insects between individuals and their external environment, and individuals locates their partners and host plants by using their sensilla. In order to achieve successful control of agricultural pests using synthetic sex pheromones, it is essential to have a better understanding of the peripheral sensory structure involved in the perception of pheromones, especially those of the antenna. Therefore, the studying of chemoreceptor’s sensilla can be useful for controlling these destructive flies by using insecticides that block the function of these sensilla.
